# Energy intake, hydration status, and sleep of world-class male archers during competition

**DOI:** 10.1080/15502783.2024.2345358

**Published:** 2024-05-06

**Authors:** Ozcan Esen, Ian Walshe, Stuart Goodall

**Affiliations:** a Northumbria University, Department of Sport, Exercise & Rehabilitation, Faculty of Health and Life Sciences, Newcastle upon Tyne, UK; b North-West University Potchefstroom, Physical Activity, Sport and Recreation Research Focus Area, Faculty of Health Sciences, South Africa

**Keywords:** Archery, carbohydrate, fluid, sports nutrition

## Abstract

**Background:**

Nutritional intake and sleep, play an important role for recovery and performance in elite sport but little work has been undertaken in archery. The present study aimed to assess energy intake (EI), hydration status, and sleep parameters in world-class male archers over the course of a four-day competition.

**Methods:**

Results, Conclusions Six male, elite-standard archers participated in the study and measurements of hydration status, EI, competition load, and sleep were recorded throughout each day of competition.

**Results:**

Daily energy, carbohydrate, and protein intake ranged between 2,563 and 3,986 kcal, 4 and 7.1 g/kg BM, 2.2 and 3.6 g/kg BM per day, respectively. Thus, archers practiced elements of periodized nutrition such that energy and carbohydrate intake was greater on the high-volume competition days (i.e. days 1 and 3; more numbers of arrows, longer duration, and walking distance) in comparison to low-volume days (days 2 and 4) over the tournament (all *p* > 0.01). Additionally, urine specific gravity was higher after waking, compared to pre- and post-competition, and before bed (all *p* < 0.05). This indicates that archers were euhydrated pre- and post-competition and before bedtime, while they were slightly hypohydrated after waking up. Sleep data show that disturbances were kept to a minimum.

**Conclusions:**

Collectively, archers appear capable of periodizing their nutritional intake according to daily physical loading during a tournament whilst, staying euhydrated and maintaining sleep quality. In part, such data can help to explain why these archers experience a sustained level of success.

## 1. Introduction

Recurve archery is an Olympic sport that requires intermittent repetitive bouts of shooting where precision is the ultimate key to success. Although archery is described as a mental sport due to its high psychological demands and victory and defeat are often influenced by the archers’ anxiety, tension, stress, and pressure, archery performance requires fine movement control, upper body strength, and endurance [1,2]. The average force required to pull the bow has been estimated as 20 kg for men and 18 kg for women archers [3]. Given these numbers are multiplied by numerous arrows fired in training and competition, this exhibits archery’s physiological demands, which seem rather disparate to other predominately aerobic or anaerobic sports. A typical recurve archery competition will last between three to four hours a day. The typical series of qualification rounds can last up to 90 s for individual matches shooting three arrows, and up to 180 s for individual and team matches shooting six arrows. The normal progression of a competition typically spans (I) Practice and Qualification Rounds (1st day; seeding for the main competition), and (II) Elimination Rounds (following days; multiple head-to-head matches until the field is narrowed down to the top competitors for the finals). The competition load varies during the elimination rounds; for instance, archers may have multiple matches in a day, leading to a higher workload compared to archers competing in fewer rounds. Consequently, the ratio of walking to sitting varies depending on factors such as the duration of the elimination rounds and the number of matches.

In the current preparation for Olympic athletes, nutrition is well accepted as a major part of athletes’ ability to perform and recover from exercise and competition [4]; however, reportedly, even professional athletes consume diets with inadequate energy and macronutrients [5,6]. More interestingly, no information exists in the current literature regarding energy consumption and nutritional intake of archers either in training or competition. As such, research is required to determine whether, and to what extent the nutritional intakes of archers are adequate. Nutrition periodization strategies for optimal performance, recovery, and health maintenance has recently been also suggested due to the apparent daily fluctuations in exercise-related energy expenditure [7]. Considering the characteristics of elite archery events, variations in dietary intake in response to changes in the total daily energy expenditure (TEE) might be an important aspect for athletes during competition. Evaluation of day-specific dietary intake throughout a tournament could elucidate the actual condition and challenges inherent to archers’ nutritional habits during competition and could reveal previously unaddressed nutritional requirements for this sport.

Hydration is another important consideration in archery performance as dehydration is known to lead to altered muscular endurance, postural control, and muscle tremor; something that euhydration can offset [8]. Archer’s experience increased parasympathetic nervous activity along with altered emotional state to sustain quality of shooting for hours during training and competition [9,10]. Indeed, increased heart rate was reported to augment tremor during rifle shooting in biathlon [11] and related to attenuated archery performance [9,12]. Further, it has been reported that heart rate was higher in archers during a competition simulation when they were dehydrated compared to euhydrated [13]. Taken together, these findings indicate that hydration status is important for archery performance and recovery, particularly during a tournament. However, to date, no study has investigated hydration status of archers either around a competition (i.e. pre- and post-competition) or throughout a day. This is important to evaluate as it could provide an archery-specific practical hydration strategy for maintaining shooting quality and/or improving archery performance.

Additionally, sleep plays an important role for both recovery and performance in elite sport. Sleep is vital for optimal immune function and endocrine function that is required for recovery from exercise training [14]. Furthermore, insufficient sleep can lead to impaired cognitive performance such as sustained attention [15], psychomotor performance [16], as well as sport-specific accuracy skills [17]. However, while it is well-established that sufficient sleep is required for optimal performance, half of elite athletes report difficulties sleeping [18], possibly due to high training loads, travel [19], and factors associated with competition [9]. Athletes often experience sleep disturbances leading into, and during competitions [20], with pre-competition anxiety being responsible for the difficulties in sleeping. However, whilst sleep data on athletes exist for several sports, less is known about sleep in elite archers during competition.

The present study aimed to simultaneously assess energy intake and hydration status in addition to sleep parameters in world-class male archers. Accordingly, a cohort of elite male archers from the Turkish National Team were studied during the European Cup in which four competition days were completed.

## 2. Methods

### 2.1. Participants

Six male, elite-standard archers from the Turkish National Archery Team (mean ± SD, age: 20 ± 2 y, body mass: 67.5 ± 6.7 kg, stature: 180.3 ± 6.2 cm, body fat: 8.5 ± 3%, V${\dot{O}}_{2}$_max_: 45.0 ± 3.3 mL·kg^−1^·min^−1^) participated in the present study. Participants included tier 5 (e.g. Olympian and Olympic or World or European Championship medalists, *n* = 3) and tier 4 athletes (e.g. international athletes, *n* = 3) [21]. All archers were involved in a regular training program at the Turkish Archery Federation Performance Centre focusing, with an average of 12 × 2.5 hours archery-based and 6 × 1 h strength and conditioning-based training sessions a week. Following explanation of the study details, all archers completed written informed consent. The study was approved by the Research Ethics Committee of Northumbria University (Reference no: 4243).

## 3. Experimental design

Measurements were taken consistently over four days during a European Grand Prix in the month of April (2023). Daily competition schedule varied (see Table 1) and took place on an outdoor grass pitch. Mean and SD of ambient temperature, humidity, pressure, and wind speed during the tournament were 14.5 ± 0.5°C, 47.3 ± 7.6%, 1,022 ± 3 mbar, and 6.5 ± 1.8 mph, respectively. The diet during tournament was nutritionist-led, with no interference from the research team.

**Table 1.** Match load variables and predicted daily total energy expenditure (TEE) (representative of average daily data from archers).

	Day 1	Day 2	Day 3	Day 4
Arrow no	180 ± 6	38 ± 5	162 ± 11	72 ± 18
Duration (min)	335 ± 22	27 ± 3	212 ± 13	113 ± 35
Walking Distance (m)	4,200	1,260	3,360 ± 543	2,590 ± 902
Predicted TEE (kcal)	4,067 ± 354	2,527 ± 203	3,467 ± 295	2,964 ± 246

## 4. Quantification of competition load

Competition and training/warm-up sessions were observed and recorded. Due to the rules during archery competitions, archers were not allowed to use any electronic/computerized monitoring devices. Variables from the competition data that were selected for analysis included number of arrows shot, duration of matches, and total walking distance covered. Duration of competition is defined from the official start time of the competition for each day, to the end time of the competition for each archer (including the number of matches, walking between rounds, and waiting between rounds and matches). The walking distance covered between rounds (archers shoot from 70 m distance and thus walk ~140 m after each series) was recorded per each match and quantify after completing the competition of the day per each archer. Archers’ daily energy expenditure was calculated by estimated resting metabolic rate [22] and the metabolic equivalent of task during competition [23]. The most recent American College Sports Medicine Position Stands recommended the Harris-Benedict equations for estimating resting metabolic rate in the athletic population [4], which seemed to be the closest estimate for male athletes [24].

## 5. Assessment of energy intake

Archers’ dietary intake at meals and snacks throughout a day and around competitions (before, during, after) was recorded via food diaries. Archers were already familiar with completing food diary entries and the lead researcher explained it on the day preceding the tournament. Each meal was also photographed by the lead researcher to obtain a better understanding of portion size and amount of food that players may have missed on their food diary entry. This type of method has been shown to accurately measure the energy intake (EI) of free-living individuals [25]. The reliability of this approach is enhanced by using food diaries and photographs which were reviewed and cross-checked [26]. Archers were provided with breakfast and dinner at the team hotel, whilst lunch was provided the nearby competition ground. For meals provided at the hotel and competition ground, menus were provided on a set menu. During the tournament, the qualified sports nutritionist prescribed main meals by using the validated the Athlete’s Plate Nutrition Educational Tool [27] but did not provide macronutrients and calorie composition. The same nutritionist has provided nutrition education and recommendations (e.g. optimizing nutritional intake around training and competition) throughout the season. Archers’ nutrition intake during the competition (e.g. carbohydrate sources such as gels, sports drinks, granola, and protein sources [protein bar, powder, and nuts]) was also planned by the same sports nutritionist on the basis of current generic sports nutrition guidelines [4]. All meals were consumed ad libitum and it was not compulsory to eat or/and finish the meals provided at the competition ground or hotel. Diaries for each meal and snack were subsequently analyzed using professional dietary analysis software (Nutritics Ltd, Ireland). Energy intake was reported in kilocalories (kcal) with macronutrient intakes analyzed and reported in grams (g), and grams per kilogram of body mass (g/kg BM).

## 6. Assessment of hydration status

Urine samples after waking up, pre- (~30 min before) and post-competition (~15 min after) and before (30–60 min before) bedtime were collected from all archers. Samples were subsequently analyzed using a handheld refractometer (Atago 3730 Pen-Pro Dip-Style Digital Refractometer, Washington, USA) to provide an indication of hydration status throughout the day. Hydration status was classified as follows: euhydrated (Urine Specific Gravity [USG] <1.020), minimally hypo-hydrated (USG 1.020–1.024) and hypo-hydrated (USG >1.024) [4]. Urine color (UC) was also recorded, by the same investigator, using a urine color chart, with numbers between 1 and 3 (very pale to pale, euhydration) and 5–8 (dark to very dark, hypohydration) [28].

## 7. Sleep

Sleep was assessed using the consensus sleep diary (core) [29], and furthermore, sleepiness was assessed using the Karolinska Sleepiness Scale [30]. Both were completed 30 minutes after awakening. Objective sleep time was assessed using actigraphy monitors worn on the wrist (GeneActiv, Cambridgeshire, United Kingdom). Given that watches were not permitted to be worn during competition, Archers were instructed to wear the monitors prior to going to bed. Participants pressed a button on the actigraphy monitor to yield a time stamped marker to indicate when they were attempting to fall asleep and remained wearing the watch until morning, where a time-stamped marker indicated their final awakening. Data from the actigraphy monitors were sampled at 10 Hz and assessed at 60-second epochs as described previously [31]. Data extracted included total sleep time (using sleep diaries to help corroborate and inform sleep data from the actigraphy monitors), while data extracted from the sleep diaries included sleep latency and wake after sleep onset. The Karolinska Sleepiness Scale provided details of subjective sleepiness after wakening. This scale includes values with an accompanied description, ranging from 1 – Extremely alert, to 10 – Extremely sleepy, falling asleep.

## 8. Statistical analysis

Normality of all data was confirmed via the Shapiro–Wilk test and competition load data are shown for descriptive purposes only. A one-way repeated-measures ANOVA was used to evaluate changes in daily energy and macronutrient intake, hydration status, and sleep between days. Hydration status throughout the 24-h was also analyzed using a one-way repeated-measures ANOVA. When there was a significant effect of “day” in daily energy and macronutrient intake, hydration status and sleeping, and effect of “time-point” in 24-h hydration status, paired sample t-tests were applied to identify which day and which time-point differed, respectively. The magnitude of the effect sizes was measured with Cohen’s d (<0.20, 0.2–0.5, 0.5–0.8, and > 0.8 are considered as “trivial,” small,” “medium,” and “large” ES, respectively). Statistical significance was set at *p* < 0.05, and all data were analyzed using SPSS 28.0 (IBM Corp., Armonk, NY), and are presented as mean ± SD.

## 9. Results

An overview of the average competitive load and estimated daily energy expenditure are presented in Table 1.

**Figure 1.**
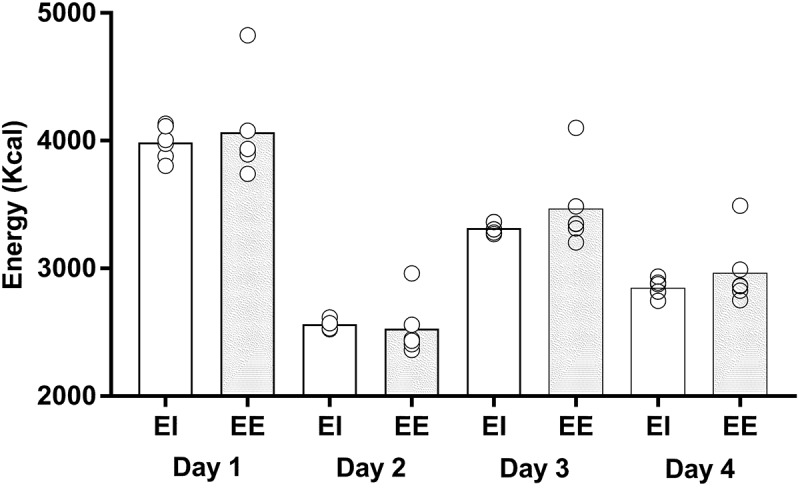
The group mean and individual energy intake (EI) and predicted daily energy expenditure (EE).

The group mean relative and absolute daily energy, carbohydrate and protein and percentage of fat intake over the 4-day tournament values are reported in Table 2. The EI and predicted EE of each individual and the group are shown in Figure 1. Daily absolute and relative EI, carbohydrate and protein intake were different across the 4-days. On day 1, absolute and relative EI were higher than days 2, 3, and 4 (all *p* < 0.001). In addition, absolute and relative EI were higher on day 3 compared to days 2 and 4 (both *p* < 0.001), and higher in day 4 compared to day 2 (*p* < 0.001).

**Table 2.** The group mean relative and absolute daily energy, carbohydrate (CHO) and protein (PRO) and percentage of fat intake over the 4-day tournament.

	Day 1	Day 2	Day 3	Day 4
EI_absolute_ (kcal)	3985 ± 118^bcd^	2564 ± 31	3315 ± 37^bd^	2847 ± 61
EI_relative_ (kcal/kg·day)	60 ± 5^bcd^	38 ± 3	50 ± 4^bd^	43 ± 4
CHO_absolute_ (g)	473 ± 24^bcd^	268 ± 8	386 ± 7^d^	386 ± 16^b^
CHO_relative_ (g/kg BM)	7.1 ± 0.6^bcd^	4.0 ± 0.4	5.8 ± 0.5^d^	5.8 ± 0.5^b^
PRO _absolute_ (g)	239 ± 6^bd^	147 ± 4	230 ± 2^bd^	147 + 18
PRO _relative_ (g/kg BM)	3.6 ± 0.3^bd^	2.2 ± 0.2	3.6 ± 0.3^bd^	2.2 ± 0.4
Fat (%)	28.4 ± 0.5^bc^	36.3 ± 0.4^acd^	24.4 ± 0.2	24.6 ± 0.5

bcd: day 1 is significantly higher than day 2, 3 and 4; bd: day 3 is significantly higher than day 2 and 4; b: day 4 is significantly higher than day 2; d: day 3 is significantly higher than day 2.

Absolute and relative carbohydrate intakes were higher on day 1 compared to days 2, 3, and 4 (all *p* < 0.01). Additionally, absolute and relative carbohydrate intake were higher on days 3 and 4 compared to day 2 (both *p* < 0.01), but there was no difference between days 3 and 4 (*p* > 0.05). In relation to protein, both absolute and relative intake were greater on days 1 and 3 compared to days 2 and 4 (all *p* < 0.001); however, there was no difference between days 1 and 3, and days 2 and 4 (all *p* > 0.05). Percentage of fat intake was higher on day 1 compared to days 4 and 3, but lower compared to day 2 (all *p* < 0.001). On days 4 and 3, fat intake was similar (*p* > 0.05), but lower compared to day 2 (both *p* > 0.05)

**Figure 2.**
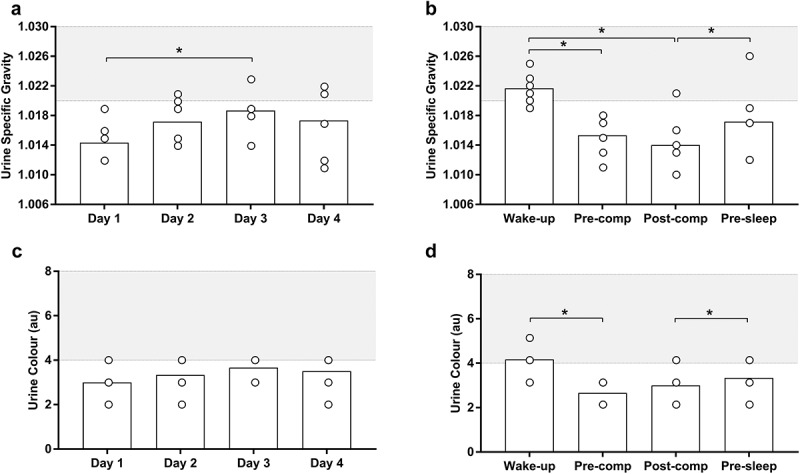
Urine specific gravity (USG) values for 24-h across 4-days (2a) and at different time points during a day (2b). UC rating for 24-h across 4-days (2c) and at different time points during a day (2d). *indicates significant differences between days and time-points (*p* < 0.05). White circles: individual values; white bars: mean values; gray shadow: minimal threshold that dehydration starts (USG >1.020).

The group mean and individuals USG and UC are shown in Figure 2. USG results for 24-h were different across the days (Figure 2(a)). Specifically, USG was higher on day 3 compared to day 1 (*p* = 0.037). Additionally, USG results were different across time-points throughout day (Figure 2(b)). Specifically, USG was higher after waking up in comparison to pre- (*p* = 0.004) and post-competition (*p* = 0.046), and higher before bed compared to post-competition (*p* = 0.007). In relation to UC, there was no difference between days (*p* = 0.058, Figure 2(c)). UC rating was different across the time-points throughout the day (*p* = 0.016, Figure 2(d)). Specifically, UC rating was higher after waking up compared to pre-competition (*p* = 0.001), and higher before bed compared to post-competition (*p* = 0.042). Those USG and UC results indicated that archers were euhydrated pre- and post-competition, and before bed, but minimally hypohydrated after waking up.

Sleep parameters are presented in Table 3. Total sleep time did not differ across the four days, however, there was a tendency for shorter total sleep time on Day 1 compared to the following days (*p* = 0.07). Subjective sleep parameters were similar across the four-day period, including sleep onset latency (*p* = 0.59) and wake after sleep onset (*p* = 0.93). Sleepiness also showed no differences across the four days (*p* = 0.47).

**Table 3.** Sleep parameters across the four-day tournament. Data presented as mean ± standard deviation. KSS – Karolinska Sleepiness Scale (10 = extremely sleepy, falling asleep).

	Day 1	Day 2	Day 3	Day 4
Total Sleep Time (min)	465 ± 38	529 ± 38	529 ± 61	482 ± 29
Sleep Onset Latency (min)	8 ± 4	9 ± 4	6 ± 3	6 ± 5
Wake After Sleep Onset (min)	4 ± 7	5 ± 4	2 ± 7	4 ± 7
Sleepiness (KSS)	9 ± 1	8 ± 2	8 ± 3	9 ± 1

## 10. Discussion

This is the first study to assessed energy intake, hydration status, and sleep of world-class male archers throughout a tournament-based competition. In relation to the specific archers studied herein, the main findings suggest that (I) world-class archers’ daily energy, carbohydrate, and protein intake can range between 2,563 and 3,986 kcal, 4 and 7.1 g/kg BM, 2.2 and 3.6 g/kg BM per day, respectively. These findings indicate that archers practice elements of periodized nutrition such that energy and carbohydrate intake is greater on the high-volume competition days (i.e. more numbers of arrows, longer duration, and walking distance) in comparison to low-volume days over the tournament. This can be interpreted as a reflection of both the athlete and the nutritionist working together, highlighting that nutrition can be optimized within the confines of an archery tournament by elite archers as well as the importance of continuous education and guidance from qualified sports nutritionists. In relation to hydration, our findings show that archers were euhydrated pre- and post-competition and before bedtime while they were slightly hypohydrated after waking up. Additionally, archers were euhydrated over the 24-hour period throughout the tournament.

The mean daily EI of archers was adequate to meet their predicted daily EE in the present study suggesting that world-class elite archers are capable to matching daily energy requirement during a tournament. Carbohydrate intake was highest on day 1 (~7.1 g/kg BM), which coincided with the longest event duration, greatest walking distance, and highest number of arrows shot whereas it was lowest on day 2, when event duration was the shortest, had the lowest walking distance, and least number of arrows shot. As such, the carbohydrate intake of the archers in this study meets the current recommendations for athletes, which is 5–7 g/kg BM and 6–10 g/kg BM for athletes who performing moderate exercise for ~1 h per day and moderate- to high-intensity exercise for 1–3 h per day, respectively [4]. The total number of arrows shot depends on how early archers are eliminated or/and how far archers progress, archers would have reduced or/and cut carbohydrate intake likely due to early elimination on days 2, 3, and 4. Collectively, our findings suggest that archers seem to adopt elements of carbohydrate periodization in a two-fold manner, addressing the upcoming competition load whilst altering it based on the progress of the competition. Although it is difficult to make direct comparisons with other sports, along with the lacking literature informing the metabolic demand of an archery event, our results are partly in line with previous studies that reported adjusted EI according to training volume in elite cyclists [32], and periodized EI and carbohydrate intake according to training- and match-day in elite soccer players [33]. Future studies are required to evaluate the metabolic demand of an archery event, which would facilitate providing a more solid context for what the carbohydrate requirements are for these athletes.

The current recommended protein intake for athletes is 1.2–2.0 g/kg BM per day [4]. In the present study, protein intake was just above the higher end of these recommendations (~2.2 g/kg BM) whereas it was much higher on days 1 and 3 than these recommendations (~3.6 g/kg BM). Since it has been demonstrated that there is an established link between protein intake and cognition [34], archers in the present study might have consumed high protein to support cognitive performance in addition to repairing, remodeling, and protein turnover. Lastly, archers’ fat intake across the days (24–36%) meets the fat recommendations for athletes (20–35%) [4].

The mean USG results for 24-h were <1.020 over the 4-days in the present study suggesting that archers were euhydrated during a day throughout the tournament. Archers in the present study had a USG < 1.020 before the competition in each day, indicating that archers started the competition with euhydration. After competition, USG results were <1.020 indicating that archers consume fluid and hydrate adequately during a competition. Further, since USG results were <1.020 before bedtime, these suggest that archers are adequately re-hydrating between post-competition and bedtime. Interestingly, archers had USG > 1.020 after waking up, possibly indicative of hypohydration. Dehydration has been shown to reduce throwing accuracy in different sports such as in cricket [35] and basketball [36]. Further, it was reported that dehydration resulted in higher heart rate [13], which can increase tremors [11] and thus affect archery performance [9,12]. As such, archers and practitioners would be advised to give a particular attention on hydration strategies and routine after waking up to facilitate euhydration before competition. In relation to urine color, the findings of the present study were consistent with the USG scores for 24-h, wake-up, pre- and post-competition and before bedtime. Since urine color has been reported as a valid indicator for hydration status [28] and considering the results in the present study, it appears to be useful for archers as well. The present study assessed only USG and urine color; and since there are well- described limitations of USG for hydration evaluation [37], future research should be encouraged to assess sweat rate in addition to electrolyte loss (e.g. sodium and potassium) in warmer condition to evaluate hydration status more accurately.

Mean sleep time ranged between 465 and 529 minutes over the four-day period, suggesting that the archers were obtaining the 7–9 hours of sleep per night that is often recommended [38]. Furthermore, reported sleep onset latency and wake after sleep onset were both in line with sleep data that is often obtained from athletes [39]. However, ratings of sleepiness were typically high with average values of 8–9 over the four-day period. High sleepiness scores may indicate unrefreshed sleep which could be due altered sleep structure. While we did not assess sleep structure in this study, Leeder et al. [39] have previously highlighted that athletes typically experience more fragmented sleep when compared to non-athletes. This supports evidence of high prevalence of sleep complaints, including non-restorative sleep in elite athletes [40].

Sleep disturbance is common among elite athletes prior to competition, often with anxiety cited as being responsible for this sleep disturbance [20]. Our results indicate minimal impact on total sleep time, sleep latency and wake after sleep onset, and therefore it is likely that the athletes had low cognitive arousal before sleep. We speculate, that while the archers may experience some level of competition anxiety, it is possible that the archers may use the same techniques to improve sleep as they do within the sport in order to reduce anxiety, tension, and stress, which are associated with performance outcomes in archery. Future studies could compare the sleep parameters and sleep disturbances of target sport athletes to other sports to explore this notion further.

## 11. Practical application

According to the findings of the present study, based on the varying daily physical loading in archery during tournaments, with corresponding changes in energetic demands, world-class archers can practice elements of periodized nutrition by staying hydrated and maintaining sleep quality. Therefore, coaches, practitioners, and athletes should consider periodizing energy intake, particularly carbohydrates, day-by-day in addition to continuously checking hydration status and sleep to optimize performance during tournaments in archery. Future work in this area should simulate competition in training sessions or seek permission for responses to be collected in less official competitions, which would provide a better idea of the competition demand.

## 12. Conclusion

In conclusion, for the first time we simultaneously quantified the daily physical loading, EI and EE, of elite level archers during an international (European Cup) tournament. Archers appear capable of periodizing their EI and CHO intake according to daily physical loading during a tournament. Archers also seems capable of staying euhydrated and keeping sleep disturbance at minimal during a tournament. In part, such data can help to explain why these archers experience a sustained level of success.
